# Multiple and frequent trypanosomatid co-infections of insects: the Cuban case study

**DOI:** 10.1017/S0031182024000453

**Published:** 2024-05

**Authors:** Jan Votýpka, Šimon Zeman, Eva Stříbrná, Petr Pajer, Oldřich Bartoš, Petr Kment, Julius Lukeš, Julius Lukeš

**Affiliations:** 1 Department of Parasitology, Faculty of Science, Charles University, Prague, Czechia; 2 Institute of Parasitology, Biology Centre, Czech Academy of Sciences, České Budějovice (Budweis), Czechia; 3 Military Health Institute, Military Medical Agency, Prague, Czechia; 4 Department of Entomology, National Museum, Prague, Czechia; 5 Department of Ophthalmology, Thomayer University Hospital, Prague, Czechia; 6 Faculty of Sciences, University of South Bohemia, České Budějovice (Budweis), Czechia

**Keywords:** biodiversity, diptera, heteroptera, host specificity, multiple infections, monoxenous trypanosomatids, nanopore sequencing, phylogeny, systematics

## Abstract

Trypanosomatids are obligate parasites of animals, predominantly insects and vertebrates, and flowering plants. Monoxenous species, representing the vast majority of trypanosomatid diversity, develop in a single host, whereas dixenous species cycle between two hosts, of which primarily insect serves as a vector. To explore in-depth the diversity of insect trypanosomatids including their co-infections, sequence profiling of their 18S rRNA gene was used for true bugs (Hemiptera; 18% infection rate) and flies (Diptera; 10%) in Cuba. Out of 48 species (molecular operational taxonomic units) belonging to the genera *Vickermania* (16 spp.), *Blastocrithidia* (7), *Obscuromonas* (4), *Phytomonas* (5), *Leptomonas*/*Crithidia* (5), *Herpetomonas* (5), *Wallacemonas* (2), *Kentomonas* (1), *Angomonas* (1) and two unnamed genera (1 + 1), 38 species have been encountered for the first time. The detected *Wallacemonas* and *Angomonas* species constitute the most basal lineages of their respective genera, while *Vickermania* emerged as the most diverse group. The finding of *Leptomonas seymouri*, which is known to rarely infect humans, confirms that *Dysdercus* bugs are its natural hosts. A clear association of *Phytomonas* with the heteropteran family Pentatomidae hints at its narrow host association with the insect rather than plant hosts. With a focus on multiple infections of a single fly host, using deep Nanopore sequencing of 18S rRNA, we have identified co-infections with up to 8 trypanosomatid species. The fly midgut was usually occupied by several *Vickermania* species, while *Herpetomonas* and/or *Kentomonas* species prevailed in the hindgut. Metabarcoding was instrumental for analysing extensive co-infections and also allowed the identification of trypanosomatid lineages and genera.

## Graphical abstract

**Figure:**
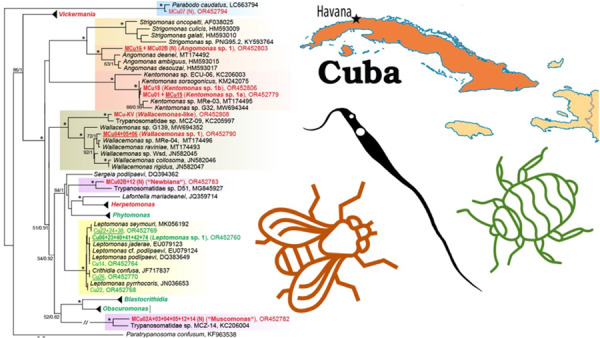

## Introduction

Until recently, trypanosomatids parasitizing insects were a relatively poorly known group of flagellates with various transmission routes (Frolov *et al*., 2021), particularly when compared to the well-studied members of the genera *Trypanosoma* and *Leishmania*, the causative agents of serious diseases of humans and other vertebrates (Maslov *et al*., 2013). However, this has now changed as trypanosomatids are turning into model organisms suitable for addressing general questions in evolutionary, cell and molecular biology (Lukeš *et al*., 2023). Many species can quickly achieve high-cell density in inexpensive media, are non-pathogenic for humans, and their assembled and annotated genomes became available (Albanaz *et al*., 2023). Moreover, they are generally amenable to genetic modifications using a battery of tools extensively used in forward and reverse genetics of *Trypanosoma brucei* and *Leishmania* species (Matthews, 2015).

Phylogenetic studies revealed that monoxenous (single-host) trypanosomatids of invertebrates, now classified into 22 genera (Kostygov *et al*., 2024), are the predecessors of dixenous (2-host) phytomonads, leishmanias, and trypanosomes (Lukeš *et al*., 2014). Therefore, the former flagellates emerged as organisms important for our understanding of complex life cycles, pathogenicity, and the unusual cell and molecular features of the latter serious human pathogens. Furthermore, trypanosomatids belonging to the genera *Angomonas*, *Strigomonas*, *Kentomonas*, *Novymonas*, and *Phytomonas* contain symbiotic bacteria that appear to represent various stages in the gradual transition of the endosymbiont into an organelle fully controlled by the host (De Souza and Motta, 1999; Kostygov *et al*., 2017; Ganyukova *et al*., 2020). Consequently, they are likely to become particularly informative for the studies of symbiosis (Husnik *et al*., 2021; Zakharova *et al*., 2023). Members of the morphologically conspicuous genus *Blastocrithidia* were shown to have a reassigned genetic code with all 3 stop codons coding for amino acids (Záhonová *et al*., 2016). Even more importantly, a novel mechanism responsible for this departure from the canonical genetic code has been discovered in *Blastocrithidia nonstop* (Kachale *et al*., 2023) and may have a wider occurrence (Baranov and Atkins, 2023).

Another interesting aspect of trypanosomatid biology is their emerging extreme diversity. Early estimates based on the ‘one-host, one-parasite paradigm’ and the observed high prevalence in the dipteran and hemipteran insects (Podlipaev *et al*., 2004) led to the prediction of over a million species (Stevens, 2001). However, more in-depth studies showed, both experimentally and under natural conditions, that this paradigm does not hold and that the same trypanosomatid species can infect several host species across continents (Kostygov *et al*., 2014; Králová *et al*., 2019; Votýpka *et al*., 2012*a*, 2020). Furthermore, insect orders other than Hemiptera, Diptera, and Siphonaptera are only rarely infected and their trypanosomatid fauna is much less speciose. Inevitably, these observations, further strengthened by the cosmopolitan presence of some trypanosomatids (e.g. *B. nonstop* and *Crithidia mellificae*) and their repeated encounters in different continents (Králová *et al*., 2019; Votýpka *et al*., 2012*a*, 2012*b*, 2020; Dario *et al*., 2021) imply that the number of extant trypanosomatid species is orders of magnitude lower.

Still, one aspect significantly complicates this picture, namely the observation that morphologically diverse flagellates are rather frequently encountered in a single insect host (Yurchenko *et al*., 2016; Frolov *et al*., 2017, 2019). This finding was further corroborated by the serendipitous observation that trypanosomatids established in culture quite often differ from those detected by sequencing in the original host (Kostygov *et al*., 2011, 2014), providing additional evidence of more than a single parasite species per host. Consequently, it can be assumed that using the PCR-based detection, genes from the most abundant flagellate species are preferentially amplified, with the co-occurring parasites going unnoticed. Since co-infections with several *Trypanosoma* species are common in *Glossina* flies, mixed infections of monoxenous trypanosomatids would not be surprising.

Therefore, we aimed to disentangle the composition of mixed trypanosomatid infections. For this, we have used a set of samples isolated from the hemipteran and dipteran insects captured in Cuba. Nanopore sequencing technology allowed us to determine that indeed, in an individual host specimen, up to 8 trypanosomatid species may co-occur. Although not species-specific and limited to a particular ecological group, when reasonably extrapolated to tropical insect diversity, such co-infections could again substantially increase the plausible species richness and host specificity of trypanosomatids, concealed by their morphological uniformity.

## Materials and methods

### Fieldwork, cultivation and host identification

True bugs and flies have been collected by using sweep netting and handpicking in several Cuban locations (Cienfuegos: 22°10'N, 80°24'W, 20 m; Las Palmas: 22°43'N, 83°32'W, 145 m; Matanzas: 23°1'N, 81°29'W, 40 m; Palma Rubia: 22°51'N, 83°27'W, 5 m; Soroa: 22°46'N, 83°0'W, 80 m; Topes de Collantes: 21°53'N, 80°1'W, 650 m; Trinidad: 21°47'N, 79°58'W, 35 m; Vinales: 22°36'N, 83°44'W, 140 m). The insects were dissected within 12 h of capture and the infected tissues were processed for DNA, smears and cultivation in the brain heart infusion medium supplemented with antibiotics, following a protocol described elsewhere (Lukeš and Votýpka, 2020). The digestive tract was gently removed in a way that did not compromise the insect except for a few abdominal segments and if possible, it was divided into midgut (mesenteron) and hindgut (proctodeum). Following the establishment of species identity of the wet or dry-mounted specimens, these have been deposited in the National Museum of the Czech Republic, Prague. Upon transport to the laboratory, an axenic culture was established from a subset of trypanosomatid-positive samples, sometimes taking up to 6 months of continuous cultivation at room temperature without shaking.

### DNA extraction, amplification and sequencing

Total genomic DNA was isolated from gut tissues or, in the case of successful cultivations, from 1 mL of axenically grown cultures, using a protocol described previously (Votýpka *et al*., 2014). To amplify the 18S rRNA trypanosomatid gene, ~10 ng of DNA was subjected to nested PCR following Seward *et al*. (2017). Sequences of PCR products obtained by Sanger sequencing were checked using Geneious software (version 10.0.6, https://www.geneious.com) and if mixed infections occurred, Oxford Nanopore Technologies (ONT) sequencing was applied, with the same total genomic DNA and outer primers as used for PCR amplification. However, the conditions were optimized for use in a one-step PCR amplification. Briefly, the PCR reaction contained TrN-F2 and TrN-R2 primers at final concentration of 400 nM each, AccuTaq polymerase (1U/25 *μ*L), PCR buffer (Sigma) supplemented with 1 M betain and 1% DMSO. The number of PCR cycles (n) ranged from 24 to 33 and has been optimized for individual samples to prevent overcycling. The PCR conditions were as follows: denaturation step 95°C for 2 min, amplification n-times (95°C for 15 s, 63°C for 30 s, and 65°C for 5 min), and final extension step at 68°C for 20 min. The extended elongation time has been important both for effective amplification and prevention of artefacts.

Subsequently, libraries for ONT sequencing were prepared from purified PCR products using a ligation sequencing (SQK-LSK109) and native barcoding expansion 1-12 and 13-24 (EXP-NBD104 and EXP-NBD114) kits, according to the manufacturer´s instructions. Libraries were sequenced on the ONT GridION platform using the R9.4 chemistry (Flow-Cell). Sequencing data were base-called, i.e. transmission from physical changes in the electric current signal measured by the ONT sequencing device to biologically relevant bases, using Guppy v.5.1.13 (Wick *et al*., 2019).

The sequencing data were processed using the Porechop v.0.2.4 pipeline to trim the barcodes and to discard possible technical chimeric reads, i.e. reads with barcode or any other technical sequence in the middle of the read (Wick *et al*., 2017). Then, we utilized the NanoCLUST pipeline to resolve the species clusters and/or representative sequences. All samples were processed using the following parameters: –min_read_length 1800 –max_read_length 2100 –cluster_sel_epsilon 1 and the limit for cluster size was set as 4% of the total reads for a given sample. When such parameters led a to crash of the pipeline in any step, we tweaked –cluster_sel_epsilon and minimal cluster size until the pipeline identified reasonable clustering within the data, based on visual inspection. In such a case, only the clusters/sequences that fulfilled the condition of cluster size = > 4% of total reads were reported. Finally, the proportional representation of the individual species clusters was estimated using a custom R script (R core team, 2020). When cultivation provided 18S rRNA sequences that were missing from the ONT sequencing, we searched for such sequences either by relaxing minimal cluster size of the NanoCLUST pipeline or by direct search of the original data using BLASTn (Camacho *et al*., 2009).

### Phylogeny

Alignments for phylogenetic analysis were generated by MAFFT v.7 using the related sequences available in GenBank of the (nearly) full-size 18S rRNA gene. The final dataset contains 2222 characters and 131 sequences representing formally described species or molecular operational taxonomic units (mOTUs), which constitute proxy (geno)species, represented in some cases by several genotypes. Phylogenetic reconstructions were performed using maximum likelihood (ML; PhyML v.3.0.1) and Bayesian inference (BI; MrBayes v.3.2.2) with model optimization in ModelTest v.3.0.6. A general time-reversible substitution model with a mixed model for the variation of site rate (GTR + Γ + I) was chosen as the best-fit model for sequence evolution. Bootstrap analyses involved heuristic searches with 1000 replicates (maximum likelihood). Bayesian inference analysis was run for 5 million generations with covarion and sampling every 100 generations. All other parameters were left in their default states.

### Nomenclature

To prevent any confusion regarding the ‘Newbiana’ and ‘Muscomonas’ nomenclature, we herein disclaim these two informal names (given in quotation marks and not italicized) for nomenclatural purposes according to The International Code of Zoological Nomenclature, Chapter 3, Article 8.3. (ICZN 1999, https://www.iczn.org/the-code/the-code-online/), thus preventing them from becoming available and entering any possible homonymy until properly described. We recommend authors using the names in question to follow the same notation method.

## Results

### Trypanosomatids infecting hemipteran hosts

Out of 417 dissected hemipterans (all from the suborder Heteroptera) belonging to 16 families and 44 species, 74 specimens (18%) were detected by microscopic examination to be infected with trypanosomatids (Tables 1 and 2). Particularly high prevalence was observed for the families Pyrrhocoridae (28 examined/10 infected/36% prevalence), Lygaeidae (56/14/25%), Rhopalidae (132/31/23%), and Alydidae (10/2/20%), with the genera *Niesthrea* (Rhopalidae), *Ochrostomus* (Lygaeidae), and *Dysdercus* (Pyrrhocoridae) accounting for 2 thirds of all positive samples. In Largidae (2/2/100%) and Reduviidae (4/2/50%) the prevalence was even higher, however, only a low number of individuals from these families was dissected.

**Table 1.** Summarized information about Cuban true bug (Heteroptera) hosts and their trypanosomatids, including the prevalence of parasites (number of dissected vs infected specimens) and the list of detected trypanosomatid species based on Sanger and Nanopore sequencing following the (nested) PCR of the homogenized host intestine and cultivation

Host family	Host species	Dissected/ infected	Detected trypanosomatid species (No. of infected specimens)
Alydidae	*Neomegalotomus rufipes*	5/2	*Phytomonas* sp. 1
	*Stenocoris filiformis*	5/0	
Aradidae	*Brachyrhynchus membranaceus*	1/0	
Coreidae	*Chariesterus gracilicornis*	1/0	
	*Spartocera batatas*	2/0	
Gerridae	sp.	2/0	
Largidae	*Largus sellatus*	2/2	*Crithidia confusa, Blastocrithidia papi/largi, Blastocrithidia* sp. 3
Lygaeidae	*Neortholomus jamaicensis*	27/0	
	*Nysius raphanus*	2/0	
	*Ochrostomus pulchellus*	16/10 (63%)	*Leptomonas* sp. 1 (10 × ), *Obscuromonas* sp. 1
	*Oncopeltus cingulifer*	7/4	*Blastocrithidia* sp. 5 (2 × ), *Obscuromonas oborniki*, *Obscuromonas* sp. 1
	*Xyonysius basalis*	4/0	
Miridae	*Collaria oleosa*	2/0	
	*Creontiades rubrinervis*	22/0	
	*Dolichomiris linearis*	25/3 (12%)	*Obscuromonas* TU18
	*Polymerus testaceipes*	12/0	
	*Taylorilygus apicalis*	2/0	
Nabidae	*Nabis capsiformis*	2/0	
Oxycarenidae	*Oxycarenus hyalinipennis*	14/0	
Pachygronthidae	*Oedancala cubana*	1/0	
Pentatomidae	*Euschistus crenator crenator*	10/1 (10%)	*Phytomonas* sp. 2
	*Chinavia rolstoni*	2/1	*Phytomonas* sp. 2
	*Mormidea angustata*	14/1 (7%)	*Blastocrithidia* sp. 1
	*Nezara viridula*	1/1	*Phytomonas* sp. 3
	*Oebalus pugnax*	9/0	
	*Oebalus ypsilongriseus*	2/0	
	*Piezodorus guildinii*	4/1	*Phytomonas* sp. 4
	*Thyanta perditor*	9/1	*Phytomonas* sp. 2
Pyrrhocoridae	*Dysdercus andreae*	7/3	*Leptomonas pyrrhocoris* (2 × ), *Leptomonas seymouri*
	*Dysdercus mimulus*	20/6 (30%)	*Leptomonas pyrrhocoris* (2 × ), *Leptomonas seymouri* (4 × ), *Blastocrithidia nonstop*
	*Dysdercus suturellus*	1/1	*Leptomonas seymouri*
Reduviidae	*Sinea* cf. *diademata*	2/1	*Blastocrithidia papi/largi*
	*Zelus longipes*	2/1	*Blastocrithidia* sp. 2
Rhopalidae	*Harmostes serratus*	6/0	
	*Jadera sanguinolenta*	5/2	*Leptomonas podlipaevi*, *Blastocrithidia* sp. 1
	*Liorhyssus hyalinus*	4/1	*Obscuromonas* TU18, *Obscuromonas* TU73b
	*Niesthrea flava*	30/17 (57%)	*Phytomonas* sp. 1 (17 × ), *Obscuromonas* TU73b (3 × )
	*Niesthrea sidae*	87/12 (14%)	*Leptomonas* sp. 1 (2 × ), *Phytomonas* sp. 1 (9 × ), *Blastocrithidia nonstop*, *Obscuromonas* TU73b (3 × )
Rhyparochromidae	*Neopamera bilobata*	3/0	
	*Ozophora pallescens*	1/0	
	*Paromius longulus*	19/1 (5%)	*Blastocrithidia* sp. 4
Scutelleridae	*Diolcus variegatus*	22/1 (5%)	*Blastocrithidia* sp. 3
	*Sphyrocoris obliquus*	3/1	*Blastocrithidia papi/largi*

**Table 2.** Summarized information about the detected trypanosomatids in Heteroptera insect host species (including number of infected specimens), localization of the infection in the host intestine and availability in culture

Trypanosomatid species	Host species (no. of infected specimens)	Tissue*	Culture
*Leptomonas podlipaevi*	*Jadera sanguinolenta*	MG	
*Leptomonas pyrrhocoris*	*Dysdercus andreae* (2 × ), *Dysdercus mimulus* (2 × )	MG	
*Leptomonas seymouri*	*Dysdercus mimulus* (4 × ), *Dysdercus andreae, Dysdercus suturellus*	MG (HG)	YES
*Leptomonas* sp. 1	*Ochrostomus pulchellus* (10 × ), *Niesthrea sidae* (2 × )	MG (HG)	YES
*Crithidia confusa*	*Largus sellatus*	MG	YES
*Phytomonas* sp. TU241	*Neomegalotomus rufipes*	MG	
*Phytomonas* sp. 1	*Niesthrea flava* (17 × ), *Niesthrea sidae* (9 × ), *Neomegalotomus rufipes* (2 × )	MG + HG	
*Phytomonas* sp. 2	*Euschistus crenator crenator, Chinavia rolstoni, Thyanta perditor*	MG (HG)	
*Phytomonas* sp. 3	*Nezara viridula*	HG (?)	
*Phytomonas* sp. 4	*Piezodorus guldinii*	HG (?)	YES
*Blastocrithidia nonstop*	*Dysdercus mimulus, Niesthrea sidae*	HG	
*Blastocrithidia papi/largi*	*Largus sellatus, Sinea* cf. *diademata, Sphyrocoris obliquus*	HG (MG)	
*Blastocrithidia* sp. 1	*Jadera sanguinolenta, Mormidea angustata*	(MG)	
*Blastocrithidia* sp. 2	*Zelus longipes*	MG	
*Blastocrithidia* sp. 3	*Diolcus variegatus, Largus sellatus*	MG	
*Blastocrithidia* sp. 4	*Paromius longulus*	MG	
*Blastocrithidia* sp. 5	*Oncopeltus cingulifer* (2 × )	HG	
*Obscuromonas oborniki*	*Oncopeltus cingulifer*	?	YES
*Obscuromonas* sp. TU73	*Niesthrea flava* (3 × ), *Niesthrea sidae* (3 × ), *Liorhyssus hyalinus*	HG (?)	
*Obscuromonas* sp. TU18	*Dolichomiris linearis* (3 × ), *Liorhyssus hyalinus*	MG (HG)	YES
*Obscuromonas* sp. 1	*Ochrostomus pulchellus, Oncopeltus cingulifer*	HG	

* Localization in host: HG, hindgut; MG, midgut; less infected part is in brackets.

Based on nested PCR and subsequent Sanger sequencing, the presence of trypanosomatids has been confirmed in all microscopically infected specimens (Tables 1 and 2). Co-infection of two trypanosomatid species occurred in 10 cases (13.5%), of which 8 were confirmed using ONT sequencing. Most of the mixed infections were confined to the family Rhopalidae (7 cases), but this is likely due to the large number of dissected individuals. The most common combination of trypanosomatid parasites, documented in 4 cases, was that of members of the genera *Phytomonas* and *Obscuromonas*.

**Figure 1.**
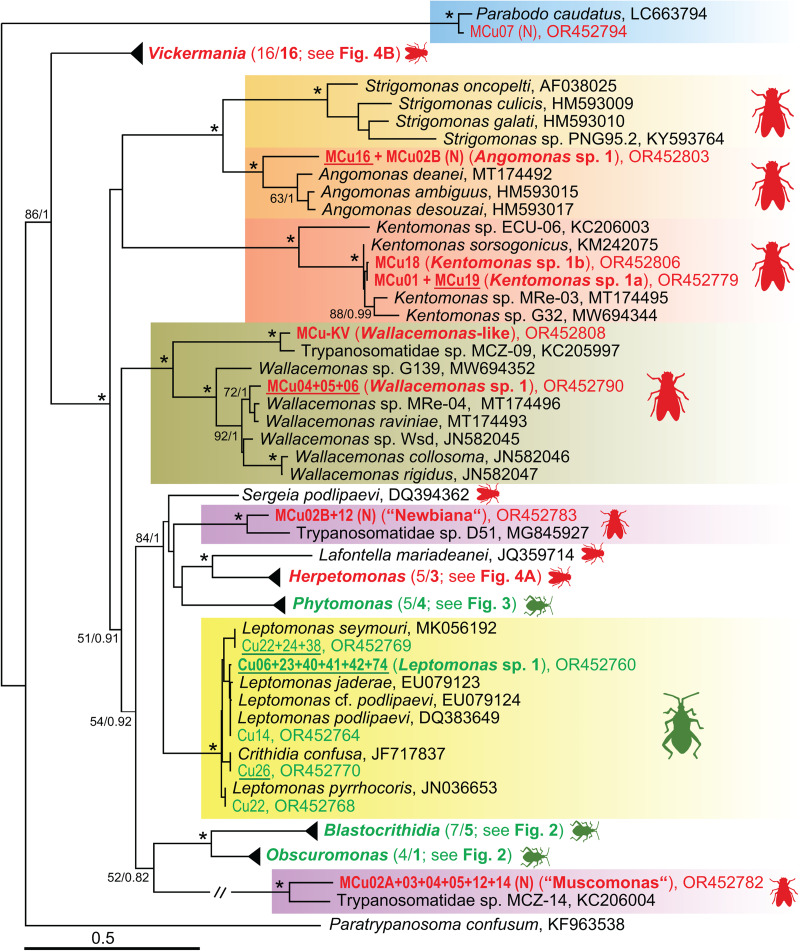
An 18S rRNA-based maximum likelihood phylogenetic reconstruction; sequences from Cuban heteropteran bugs (Cu) are indicated by green, from Cuban dipteran flies (MCu) by red, new species (mOTUs, molecular operational taxonomic units) by bold; isolates with culture established are underlined, (N) indicates detection only by Nanopore sequencing; for details of 5 selected genera see the individual subtrees (Figs 2–4, the number in brackets indicates the number of detected/number of new (in bold) mOTUs); asterisks mark branches with maximal statistical support (ML > 95, Bayesian > 0.95); double crossed branch is 50% of the original length; the scale bar denotes the number of substitutions per site.

**Figure 2.**
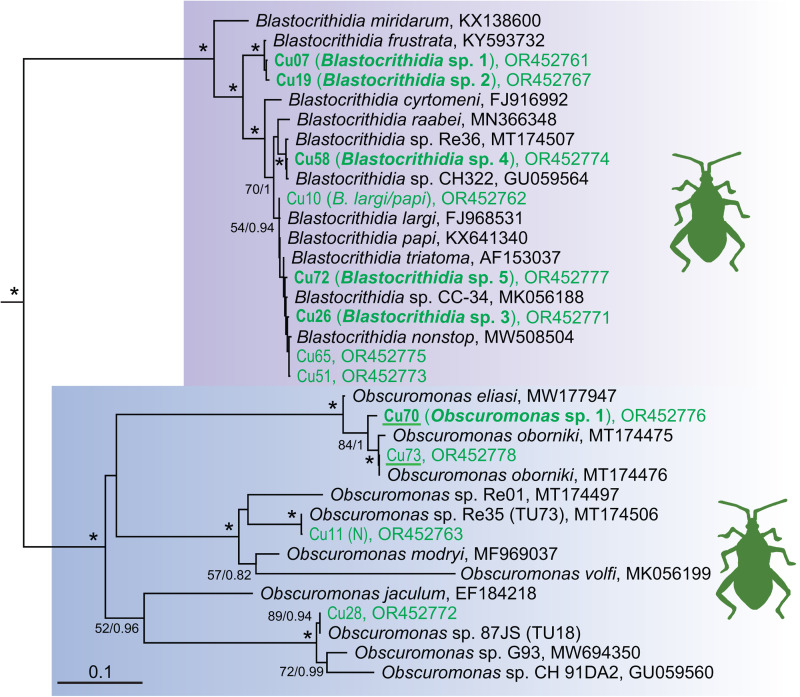
Expanded subtree of the genus *Blastocrithidia* and *Obscuromonas*; for more detail see Fig. 1.

**Figure 3.**
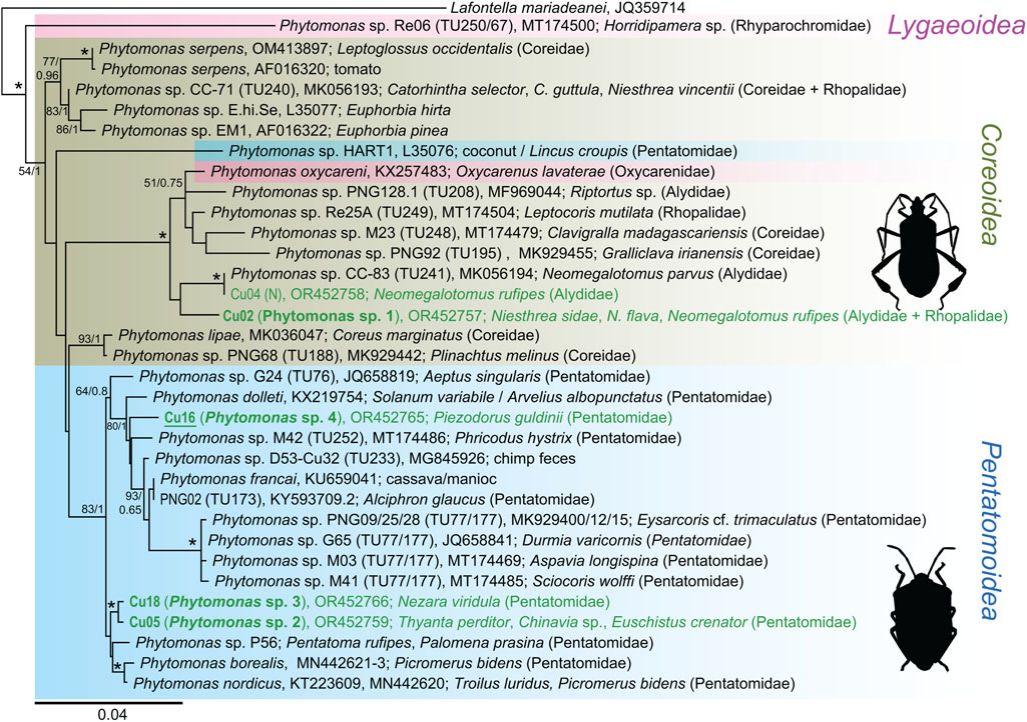
An 18S rRNA-based maximum likelihood phylogenetic reconstruction of the genus *Phytomonas* specifying the insect hosts (vectors); the blue undercolouring indicates species that are exclusively associated with the family Pentatomidae; for more detail see Fig. 1.

In total, 21 different trypanosomatid species (due to missing formal descriptions, we refer to them here as molecular operational taxonomic units [mOTUs]) were found in the examined hemipterans, of which 11 were detected for the first time. We label them as new species based on differences in the 18S rRNA sequences considered as significant for this group of flagellates. However, all detected mOTUs from the hemipteran hosts can be affiliated with already known taxa and thus do not represent any major novel lineages or genera (Figs 1–3). More than half of the new mOTUs belong to the subfamily Blastocrithidiinae, with 5 falling into the genus *Blastocrithidia*. While *Blastocrithidia* sp. 4 is closely related to (or possibly conspecific with) the isolate CH322 from China, the other 4 sequences clearly represent new species, substantially extending the known diversity of this genetically remarkable genus. Since none of the new species was found in more than 2 hemipterans (Table 2), information on their host specificity remains limited. On the other hand, several widely distributed generalist species, such as *B. nonstop*, the *Blastocrithidia papi*/*largi* species complex and *Obscuromonas oborniki*, were detected in several hemipteran families and hence possess a wide host range. Although *Obscuromonas* sp. TU73b was found in 7 bugs from the family Rhopalidae, it was invariably present in co-infections with other trypanosomatids, be it members of the genera *Phytomonas*, *Leptomonas* or other *Obscuromonas* species.

From the subfamily Herpetomonadinae, only the genus *Phytomonas* was present in the examined hemipterans (Figs 1 and 3). Four new mOTUs could be distinguished, although *Phytomonas* spp. 2 and 3, both from various shield bugs (Pentatomidae), might be considered conspecific, as their 18S rRNA genes differ in only 4 nucleotides. *Phytomonas* sp. 1 was repeatedly found in *Niesthrea* bugs (Rhopalidae) from several localities, making it the most encountered trypanosomatid in our survey. In one locality, this flagellate infected 2 individuals of *Neomegalotomus rufipes* (Alydidae), out of which one was coinfected with an already known *Phytomonas* sp. TU241 (Fig. 3, Table 2).

Of the 5 mOTUs detected within the subfamily Leishmaniinae, 4 could be assigned to already known species, with only 1 being novel (Fig. 1). *Leptomonas* sp. 1 was quite abundant and confined mostly to a single-host species, *Ochrostomus pulchellus* (Lygaeidae). While *Leptomonas podlipaevi* was detected only in one individual of Rhopalidae, *Leptomonas pyrrhocoris*, a cosmopolitan specialist associated with fire bugs (Pyrrhocoridae), as well as *Leptomonas seymouri*, were repeatedly found in several *Dysdercus* species. Finally, *Crithidia confusa* was encountered in a single co-infection with *Blastocrithidia*, being detected only in an established culture.

### Trypanosomatids infecting dipteran hosts

Of 201 dissected fly specimens, only 20 (10%) were detected by microscopic examination to be infected with trypanosomatid parasites. Of these, nested PCR and subsequent Sanger sequencing revealed co-infection in 11 cases (55%), of which all were confirmed by ONT sequencing (Table S1).

Out of 20 microscopically infected flies, 10 and 5 were members of Muscidae and Sepsidae, respectively, while the remaining 5 infected flies belonged to the families Ulidiidae (2 flies), Calliphoridae, Drosophilidae and Lauxaniidae. Since uninfected flies were not taxonomically examined, the prevalence in individual families cannot be established.

**Figure 4.**
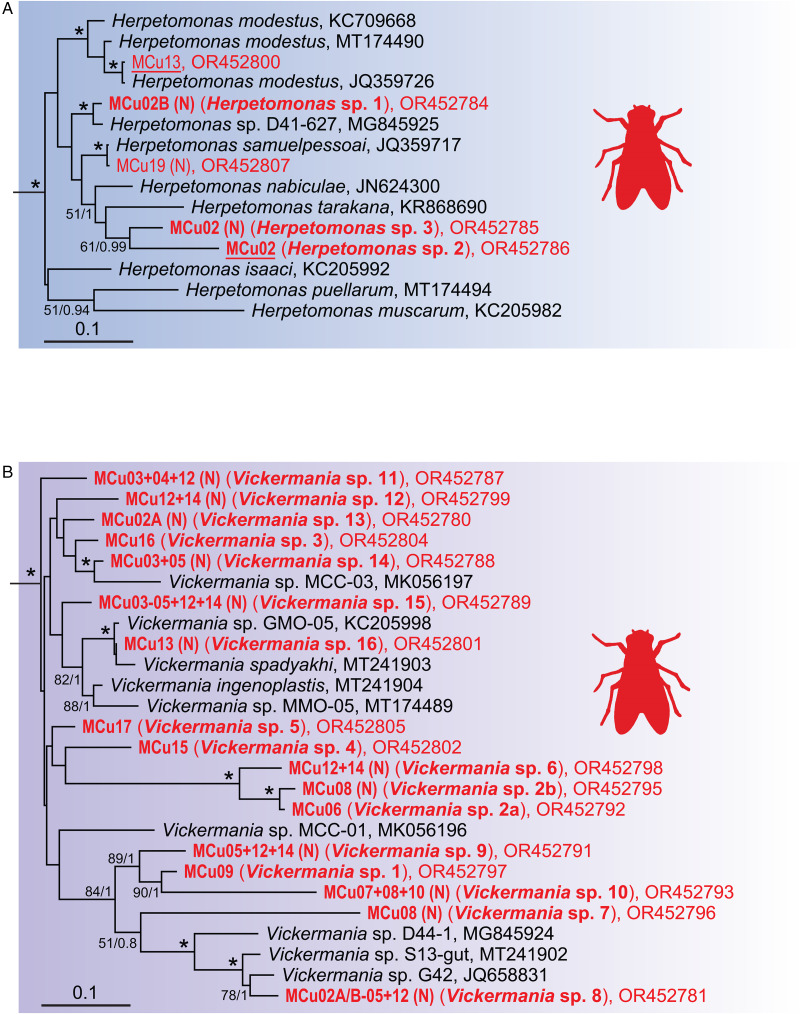
Expanded subtree of the genus *Herpetomonas* (A) and *Vickermania* (B); for more detail see Fig. 1.

Interestingly, 14 of the infected individuals (mostly belonging to Muscidae and Sepsidae) were inhabited by multiple trypanosomatid or bodonid species. In 11 cases, we were able to ascertain by deep ONT sequencing the identity of almost all trypanosomatids present in a single host, allowing us to document up to 8 species in one host specimen (MCu02 and MCu12) (Table S1, Figs 1, 3, 4 and S1). As a result, a total of 27 mOTUs were recognized, of which 25 can be considered as new species. Among them, 2 species seem to be so distant from their known relatives that their accommodation into new genera would be justifiable (see below). Trypanosomatids of the subfamily Herpetomonadinae were represented in our dataset by 5 mOTUs confined to Muscidae. Two of them, each found in one individual only, can be assigned to *Herpetomonas samuelpessoai* and *H*. *modestus* (Fig. 4A). While *Herpetomonas* sp. 2, found in 2 samples, is clearly a distinct species, the status of *Herpetomonas* spp. 1 and 3, present only in a single examined host, remains uncertain.

The symbiont-containing subfamily Strigomonadinae comprises 2 new mOTUs. Since we succeeded in introducing both into the culture, they will be subject to a detailed examination in the future. *Angomonas* sp. 1 was present in 2 individuals of Calliphoridae and Muscidae and constitutes a basal lineage of the genus (Fig. 1). Two genotypes of *Kentomonas* sp. 1, related to *K*. *sorsogonicus* (Fig. 1), were identified in 2 Muscidae and one Ulidiidae flies, respectively. Two mOTUs fall into the genus *Wallacemonas*. Being a typical member of the genus, *Wallacemonas* sp. 1 was detected in 3 flies (2 individuals from Muscidae and 1 from Ulidiidae) (Table S1, Fig. 1). On the contrary, the isolate MCu-KV, which originated from Drosophilidae, is closely related to MCZ-09 from Lauxaniidae and either forms a basal lineage of the genus or even qualifies, together with MCZ-09, as a candidate for a new genus (Fig. 1).

The surprisingly high diversity of the genus *Vickermania* (sensu Kostygov *et al*., 2020) revealed 16 mOTUs (Fig. 4B), all hitherto unknown. Most of them exhibit some level of host specificity, with *Vickermania* sp. 3 being confined to Calliphoridae, *Vickermania* sp. 4 to Lauxaniidae, *Vickermania* spp. 1, 7, and 10 to Sepsidae and, finally, *Vickermania* spp. 8, 9, 11 through 16 to Muscidae. Only *Vickermania* sp. 2 parasitized both Sepsidae and Ulidiidae, but even then, it was represented by 2 different genotypes, which could also be considered as 2 different mOTUs and therefore species. While the prevalence of members of the genus *Vickermania* was generally low, with only half of the mOTUs found in more than a single specimen, their co-infections were rather frequent in flies belonging to Muscidae and Sepsidae. As a result, 12 out of 16 mOTUs could only be detected by ONT sequencing, being masked in standard PCR by a more abundant flagellate (Table S1, Fig. 4B).

Moreover, application of this technology also allowed the detection of trypanosomatids that, based on the 18S rRNA sequences, represent 2 candidate new genera, both with unstable position in the phylogenetic tree. The first lineage, here tentatively named ‘Muscomonas’, was detected during this survey in 6 individuals of Muscidae, invariably in a co-infection with *Vickermania* spp. and/or another trypanosomatid. This candidate for a new genus is related to MCZ-14 from Opomyzidae captured in the Czech Republic (Fig. 1). The second group, here informally named ‘Newbiana’ (based on its provisional name ‘New-B’), was found in two individuals of Muscidae, with the most closely related sequence coming from a chimpanzee feacal sample from Cameroon (Fig. 1), although it has been proposed that the detected trypanosomatid originates from a fly that contaminated the sample (Votýpka *et al*., 2018). In both cases, only sequences are available in the absence of any morphological data, because in the dry smears prepared from the infected insects (data not shown), it is technically challenging to associate a given cell with a given sequence.

Finally, in one Sepsidae (MCu-07), *Parabodo caudatus* was detected (Fig. 1), while another specimen from the same family (MCu-10) carried even 3 mOTUs belonging to the genus *Parabodo* (Table S1), representing likely a passive passage of free-living flagellates from water.

### Trypanosomatid co-infections in dipteran hosts

The ONT sequencing enabled the identification of multiple trypanosomatid species co-infecting a single host, as well as the estimation of how numerous each species was. Furthermore, in some Muscidae, the midgut and the hindgut were dissected, microscopically examined and further processed separately, allowing to compare the occurrence and abundance of each trypanosomatid species in different parts of the digestive system.

**Figure 5.**
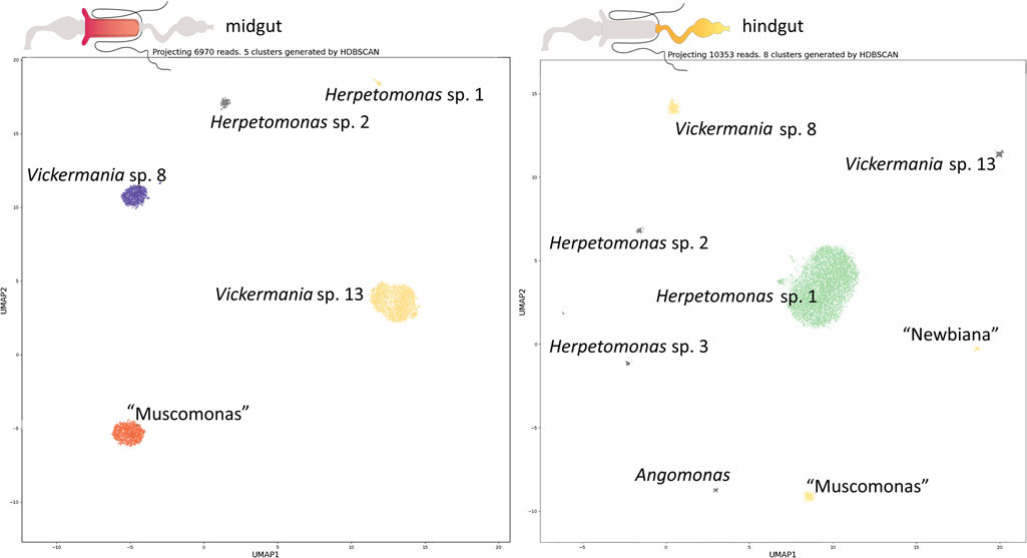
Both microscopically positive parts of the digestive tract of the muscid fly MCu02 were processed separately. By nanopore sequencing, 5 trypanosomatid species were detected in the midgut (left). While 2 *Vickermania* species (*Vickermania* sp. 8 and sp. 13) and new genus ‘Muscomonas’ are dominant inhabitants of the midgut, 2 *Herpetomonas* species (*Herpetomonas* sp. 1 and sp. 2) likely represent contaminants from the hindgut or passively passaged cells. In the hindgut (right), 8 trypanosomatids have been detected, of which *Herpetomonas* sp. 1 is the predominant species. Sequences derived from other species occur only in very low numbers and represent either cells released from the anterior part of the digestive tract (*Vickermania* sp. 8 and sp. 13 and ‘Muscomonas’) or cells that have been only passively passaged. Alternatively, they have been outcompeted by the dominant *Herpetomonas* species (in case of ‘Newbiana’, *Herpetomonas* sp. 2 and sp. 3, and *Angomonas*).

The fly midgut was generally dominated by several *Vickermania* species or by the ‘Muscomonas’ flagellates (Table S1). An example of a heavy co-infection of several species is shown for MCu-12 (Fig. S1) and MCu-02 (Fig. 5A). Other trypanosomatids, including several *Vickermania* species, were much less numerous, indicating either their competitive exclusion by the dominant species, or an accidental or perennial midgut infection.

In the hindgut, members of the genera *Herpetomonas* or *Kentomonas* prevailed (e.g., MCu-02B and MCu-19), while other flagellates were rare (Fig. 5B). When both segments of the digestive tract of a dissected specimen were infected simultaneously, a small number of typical midgut inhabitants, such as ‘Muscomonas’ and *Vickermania* spp., also occurred in the hindgut (Fig. 5B). The same applies *vice versa*, as some hindgut dwelling trypanosomatids, such as members of the genus *Herpetomonas*, can be found in the midgut (Fig. 5A). However, when one segment of the digestive tract was infected, only the corresponding trypanosomatids were found (Fig. S1). For example, ‘Muscomonas’ and *Vickermania* spp. were never found in flies with only their hindgut infested, and similarly, *Herpetomonas* spp. were not detected in hosts with an infection confined to the midgut.

## Discussion

While the last decade has seen a significant expansion of the known diversity of insect and plant trypanosomatids including some studies from Asia, Europe and Africa (Votýpka *et al*., 2010, 2012*a*; Lukeš *et al*., 2018; Frolov *et al*., 2019, 2020), it was continental South and Central America where most sampling occurred (Teixeira *et al*., 1997, 2011; Maslov *et al*., 2007, 2010; Borghesan *et al*., 2013; Kozminsky *et al*., 2015; Dario *et al*., 2021). However, except for a single study conducted on the island of Curacao (Votýpka *et al*., 2019), so far, no information was available on these interesting protists in the Caribbean. Here, we present such a study from Cuba, in which we have not only mapped the distribution and diversity of trypanosomatids but also applied ONT sequencing to examine their frequency and extent of co-infections within a single insect host.

### Trypanosomatid diversity in hemipteran hosts

Similar to other studies carried out elsewhere (Sbravate *et al*., 1989; Votýpka *et al*., 2012*a*, 2019, 2020; Kozminsky *et al*., 2015; Králová *et al*., 2019), the highest prevalence of trypanosomatids was detected in Pyrrhocoridae, Lygaeidae, Rhopalidae, Alydidae, Pentatomidae, Reduviidae, and Largidae, with Rhyparochromidae, Scutelleridae, and Miridae being infected only rarely. While the lack of infections in Coreidae, Gerridae, and Nabidae can be explained by the low number of dissected individuals, the negativity of Oxycarenidae was unexpected, since the European population of *Oxycarenus hyalinipennis* and *O*. *lavaterae* harbour several trypanosomatid species (Franchini, 1922; Antonucci, 1941; Seward *et al*., 2017). This may be caused by *O. hyalinipennis* being a non-native species introduced to America in 20th century (Grillo, 1993).

Based on the 18S rRNA sequences, 21 mOTUs could be distinguished in the studied bugs, 10 of which were already known from previous studies. This includes a cosmopolitan *B. nonstop*, known to have an extensively reassigned genetic code, which is able to parasitize bugs from 8 families (Králová *et al*., 2019; Kachale *et al*., 2023), now also including Rhopalidae (this study). The *Blastocrithidia papi*/*largi* species complex was retrieved from 3 different host families, one of which is the predatory assassin bug (Reduviidae), although due to the low intensity of infection, accidental transmission of the parasites from an infected prey cannot be excluded. Their close relatives *Obscuromonas* sp. 87JS (TU18) and *Obscuromonas* sp. Re35 (TU73) are, regardless of their geographic location, restricted to Miridae and mostly Rhopalidae, respectively (Westenberger *et al*., 2004; Maslov *et al*., 2007; Votýpka *et al*., 2020; this study). Not only is such a host preference unusual for Blastocrithidiinae, but it is also worth noting that the latter mOTU always occurred in co-infection with some other trypanosomatids, be it *Phytomonas*, *Leptomonas*, or other *Obscuromonas* species.

*Leptomonas seymouri* deserves particular attention, as it has been repeatedly detected in human cutaneous lesions caused by *Leishmania* spp. in India and neighbouring countries (Ghosh *et al*., 2012; Singh *et al*., 2013), and thus cannot be considered as just a frequent contaminant of laboratory cultures (Kraeva *et al*., 2015). Although originally described from the cotton stainer bug *Dysdercus suturellus* (Wallace, 1977), ever since this trypanosomatid has been found neither in *Dysdercus* nor in any other Heteroptera, leading to uncertainty about the identity of its true host (Kraeva *et al*., 2015). On the other hand, experimental infections were much more successful in *Dysdercus* (Moraes *et al*., 1994) than in sand flies (*Phlebotomus* spp.), the putative vector of *L. seymouri* in human lesions (Kraeva *et al*., 2015). However, our current finding puts this problem to rest, confirming that *L*. *seymouri* infects *Dysdercus* under natural conditions. Specifically, its current distribution in its insect host is confined to the Americas (Wallace, 1977; Votýpka *et al*., 2019; this study), whereas this flagellate was detected in *Leishmania* lesions in the Old World (Ghosh *et al*., 2012). Although based on old studies (Blacklock, 1923), *Dysdercus* is able to bite humans, the co-transmission with *Leishmania* in this way is highly improbable, and the vector of *L. seymouri* in human lesions thus remains unknown.

Among the new mOTUs, *Leptomonas* sp. 1 stands out due to the high prevalence in its main host, *Ochrostomus pulchellus*. Its detection also in *Niesthrea sidae* can be explained by the feeding of both hosts on the Malvaceae plants (Baranowski and Slater, 1975). It is noteworthy that the latter host species was fairly often infected by *Phytomonas* sp. 1, which was also found in 2 specimens of *Neomegalotomus rufipes* that feeds on Fabaceae (Froeschner, 1942; Ventura *et al*., 2000*a*). Alternatively, *N. rufipes* could have obtained *Phytomonas* sp. 1 from feeding on a dead *N*. *sidae*, since necrophagy has been observed in this genus (Ventura *et al*., 2000*b*), likely resulting in non-specific infections by otherwise specialized trypanosomatids (Votýpka *et al*., 2019). Although found in only a few hosts, *Phytomonas* spp. 2, 3 and 4 belong to a clade that seems to be confined to various Pentatomidae (Fig. 3), while other *Phytomonas* spp. infect mainly bugs from the superfamily Coreoidea. Moreover, the inability of *Phytomonas* from a coreoid host to infect pentatomid bugs was recently experimentally demonstrated (Malysheva *et al*., 2023). Still, no such specificity can be observed in the plant host, as *Phytomonas* species infecting different plant families often cluster together and *vice versa* (Zanetti *et al*., 2016). Therefore, it appears that *Phytomonas* spp. are primarily specialized to the insect host, being confined to a single family or superfamily, whereas the spectrum of plant hosts can be much broader. Similar situation has been documented for the dixenous genus *Leishmania*. Indeed, some *Leishmania* species infect multiple various vertebrate host species, yet are restricted to a single insect species (Akhoundi *et al*., 2016).

### Trypanosomatid diversity in dipteran hosts

Most infected flies belong to the families Muscidae and Sepsidae, which is likely due to their aggregative feeding on various liquids from dung. In total, we have identified 27 mOTUs from the dipteran hosts, of which only two were previously detected. *Herpetomonas samuelpessoai* has originally been described from an assassin bug *Zelus leucogrammus* (Galvão *et al*., 1970), but since it was later encountered only in dipterans (Sarcophagidae, Anthomyiidae, and Muscidae) (Týč *et al*., 2013; this study), it is likely that its true hosts are various brachycerans and the assassin bug infection was accidental. The other already known species is *Herpetomonas modestus* that has so far only been found in Muscidae (Týč *et al*., 2013; this study) and Calliphoridae (Borghesan *et al*., 2013).

The trypanosomatid family Strigomonadinae invariably carries bacteria, and this symbiotic relationship is being studied in its South American isolates (Teixeira *et al*., 2011; Borghesan *et al*., 2018). Both new mOTUs also contain endosymbionts (data not shown), with one of them constituting the most basal lineage of the genus *Angomonas*. Same as other *Angomonas* species, it parasitizes the hindgut and midgut of Muscidae and Calliphoridae (Ganyukova *et al*., 2017). The second novel mOTU (*Kentomonas* sp. 1) is closely related to *K. sorsogonicus*, yet instead of infecting Sarcophagidae (Votýpka *et al*., 2014), this Cuban isolate was found in Muscidae and Ulidiidae.

The recently established genus *Vickermania* (Kostygov *et al*., 2020) accommodated only 2 species so far, both isolated from Calliphoridae and Sepsidae. Hence, the discovery herein of numerous mOTUs infecting flies from Muscidae, Sepsidae, Calliphoridae, Ulidiidae, and Lauxaniidae is surprising and indicates either a particularly extensive diversity of this genus on the island or its hitherto overlooked presence elsewhere. Indeed, most *Vickermania* mOTUs have been detected only *via* ONT sequencing, which clearly demonstrates the power and utility of this approach (with the capability to sequence full-length target genes) and indicates that the latter possibility is the case, namely that these flagellates have often been overlooked in mixed infections. From the limited dataset available, rather narrow host specificity can be inferred, as no mOTU was detected in more than one dipteran family. Such a tight association is unusual among monoxenous trypanosomatids, since other Brachycera-infecting genera, such as *Herpetomonas*, *Wallacemonas*, and *Crithidia*, can be found in several dipteran families (Borghesan *et al*., 2013; Týč *et al*., 2013). It is also surprising considering that *Vickermania spadyakhi* from Sepsidae was under experimental conditions able to infect *Lucilia* flies (Calliphoridae) (Kostygov *et al*., 2020).

Among the detected flagellates, 2 groups, provisionally labelled ‘Muscomonas’ and ‘Newbiana’, deserve in terms of their sequence divergence the status of a new genera. However, the failure to introduce them into culture and describe their morphology precludes their formal description for the time being, as there is no feasible way to associate cells from mixed infections with a given sequence. One possible approach is single-cell sequencing; however, this would be most challenging with dry smears, where usually multiple cells are in close physical contact and the DNA is of poor quality. Both unnamed genera have been detected in the midgut and hindgut of Muscidae from several Cuban localities. Similarly, 2 previously published sequences clustering with these new mOTUs (Fig. 1) also originate from flies (MCZ-14) (Týč *et al*., 2013) or are very likely derived from them (D51) (Votýpka *et al*., 2018).

### Co-infections, tissue localization and host specificity

The extremely high intraspecific and low interspecific morphological variability of trypanosomatids makes it almost impossible to distinguish *in situ* even distantly related species, genera or subfamilies (Podlipaev and Lobanov, 1996). For this reason, the infections of a single host by multiple species of trypanosomatids are very difficult to discern and were for over a century (Prowazek, 1904) responsible for frequent confusions regarding species identity, morphology and life cycles. However, since the onset of the sequencing era, reliable species delimitations and identifications became possible, revealing, among other things, the commonality of co-infections (Votýpka *et al*., 2012*a*, 2019; Lukeš *et al*., 2018; Králová *et al*., 2019). Likely used for the first time in the study of insect trypanosomatids, ONT sequencing proved to be highly sensitive, revealing frequent co-infections constituted by abundant as well as (very) rare species. Furthermore, thanks to the relative ease of amplification and the comparable copy number of rRNA genes in different trypanosomatids (Albanaz *et al*., 2023), by sequencing tens of thousands of reads, it is possible to estimate how many cells are there in a sample, thus indicating the strength of infection and identifying the (pre)dominant species. There are several other ways how to identify multiple trypanosomatid infection in insects, e.g. using PCR amplification of the spliced leader RNA gene (Kozminsky *et al*., 2015). However, in the terms of accuracy and estimation of relative quantification, ONT sequencing has obvious advantages over the PCR length-based approaches.

Within an insect host, different trypanosomatids inhabit different parts of its digestive and/or excretory tracts (Frolov *et al*., 2021), resulting in a niche partitioning during co-infection. Such differentiation can be caused by distinct features of various life cycles. For example, in the tabanid *Hybomitra solstitialis*, *Wallacemonas raviniae* builds up massive loosely attached growths on the rectal wall, while *Trypanosoma theileri* adheres tightly to the ileum using an extracellular matrix. This likely reflects the transmission of the former species among its hosts through cells leaving the digestive tract, whereas the latter flagellate has no benefit in leaving the host, as its life cycle proceeds following the ingestion of an infected tabanid (Malysheva *et al*., 2022). In another case, in the herbivorous species of true bug *Coreus marginatus*, *Phytomonas* is transmitted to the host plant *via* infected salivary glands (Frolov *et al*., 2019), while the transmission of co-infecting *Blastocrithidia* among insect hosts occurs by a cyst-like stage, which is formed in the midgut and rectum (Frolov *et al*., 2020), resulting in functional niche partitioning. Indeed, the coinfections of *Phytomonas* and *Obscuromonas* or *Blastocrithidia* appears to be common among Coreidae, Rhopalidae, Alydidae and Pentatomidae (Votýpka *et al*., 2012*a*, 2019; this study).

Moreover, a niche partition can be observed even within one segment of the digestive tract. The firebug *Pyrrhocoris apterus* is commonly co-infected by *L. pyrrhocoris* and *B. papi*, both inhabiting the midgut. While *Leptomonas* dwells exclusively in the lumen of the gut (Votýpka *et al*., 2012*b*), *Blastocrithidia* usually attaches to the epithelium (Frolov *et al*., 2017). Furthermore, to multiply and produce cyst-like amastigotes, *Blastocrithidia* moves to the Malpighian tubules, where the former species is absent (Frolov *et al*., 2018). A similar situation possibly occurs in the co-infection of *Niesthrea sidae* by *Leptomonas* sp. 1 and *Obscuromonas* sp. TU73, and *Ochrostomus pulchellus* by *Leptomonas* sp. 1 and *Obscuromonas* sp. 1 detected in this study.

One possible source to compete for (apart from nutrition) is the epithelial surface to which trypanosomatids tend to adhere. In such a case, species living freely in the lumen should have a higher capacity for co-existence, as this competition factor is excluded. Such species can be confined to the midgut, where attachment is impossible (except for Heteroptera) due to the presence of peritrophic matrix (Kostygov *et al*., 2020). Indeed, while in the hindgut of a muscid fly (MCu02) *Herpetomonas* species massively predominated, there were 3 comparably large clusters of *Vickermania* sp. 8, *Vickermania* sp. 13, and ‘Muscomonas’ in the midgut. Similarly, and not exclusively, in the midgut of another muscid fly 2 non-attaching species represented by *Vickermania* sp. 8 and ‘Muscomonas’ coexisted in approximately the same intensity of infection.

Combining several methods, 48 trypanosomatid species belonging to 11 genera were detected in true bugs and flies, confirming previous findings that these parasites are highly diverse and ubiquitous, with most genera having cosmopolitan distribution. Thanks to the use of deep ONT sequencing, we were able to detect a surprisingly high diversity of insect trypanosomatids in Cuba. Even more importantly, this approach allowed us to determine a substantial proportion of mixed infections, with up to 8 species of these flagellates infecting a single fly host. When extrapolated to the fraction of dipteran and hemipteran diversity infected by these flagellates in the tropics, the estimate of trypanosomatid diversity may be justifiably increased by up to an order of magnitude, revealing one more facet of this unique group of parasites.

## Supporting information

Votýpka et al. supplementary material 1 Votýpka et al. supplementary material

Votýpka et al. supplementary material 2 Votýpka et al. supplementary material

## Data Availability

All data is available in the manuscript or supporting material.

## Supplementary material

The supplementary material for this article can be found at https://doi.org/10.1017/S0031182024000453.
