# Supplementary figures and images for: Multiple and frequent trypanosomatid co-infections of insects: the Cuban case study
Source: Parasitology. 2024 Apr 15;151(6):567–78. doi: 10.1017/S0031182024000453 (PMC11428007; doi:10.1017/S0031182024000453)

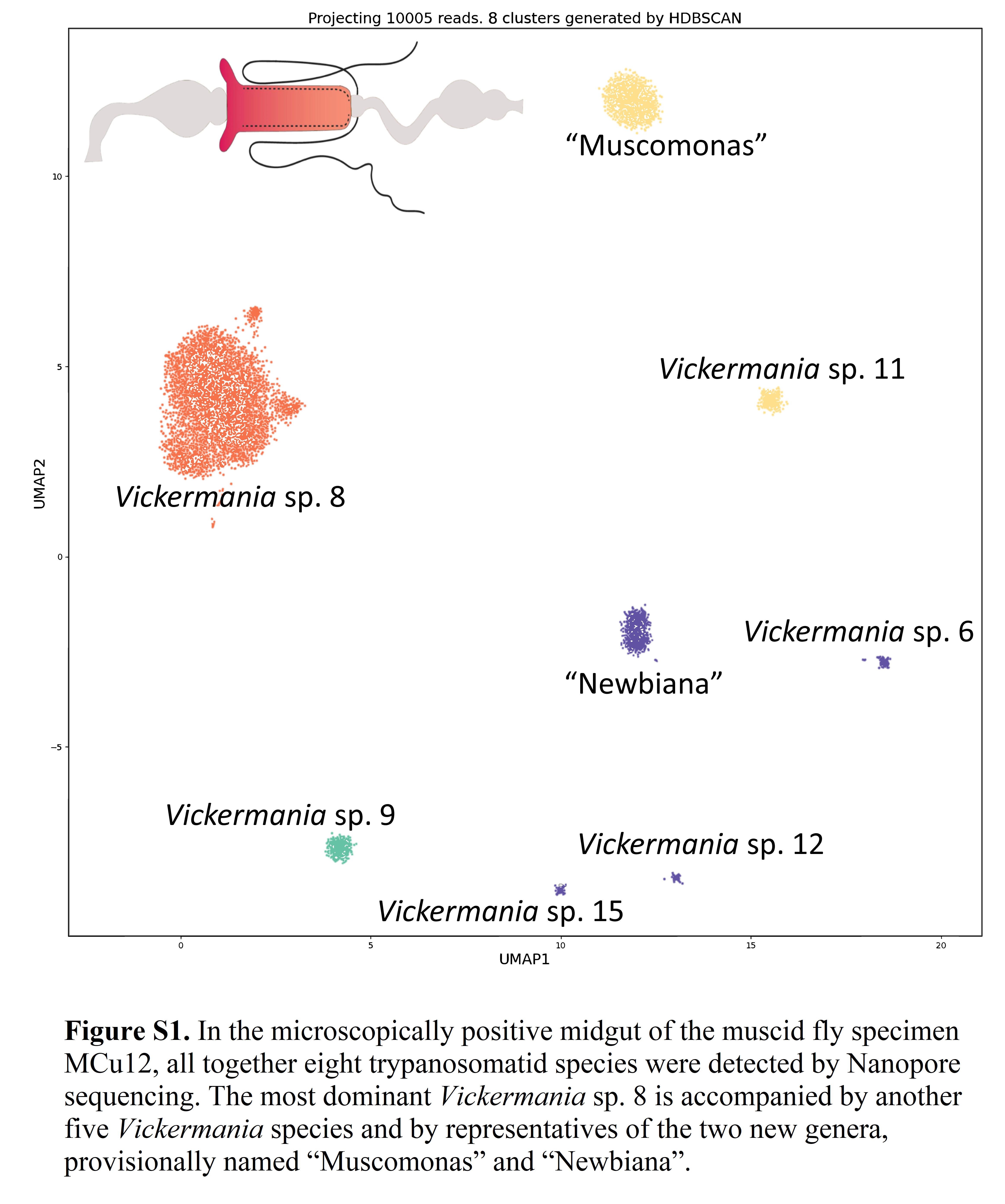

Supplement: Votýpka et al. supplementary material 1 — Votýpka et al. supplementary material [file S0031182024000453sup001.tif]
