# Supplementary material for: Multiple and frequent trypanosomatid co-infections of insects: the Cuban case study
Source: Parasitology. 2024 Apr 15;151(6):567–78. doi: 10.1017/S0031182024000453 (PMC11428007; doi:10.1017/S0031182024000453)
Supplement: Votýpka et al. supplementary material 2 — Votýpka et al. supplementary material [file S0031182024000453sup002.pdf]

Table S1. Summarized information about Cuban fly (Diptera) hosts and their trypanosomatids, including intensity of infection (**Int.**), tissue localization (**Tissue**) of the infection in the host intestine (HG, hindgut; MG, midgut) and the list of detected trypanosomatid species based on cultivation (**Culture**) and **Sanger** and/or **Nanopore** sequencing following the (nested) PCR of the homogenized host intestine.

Specimen	Locality	Family	Int.	Tissue	Culture	Sanger sequencing	Nanopor sp.#1	Nanopor sp.#2	Nanopor sp.#3	Nanopor sp.#4	Nanopor sp.#5	Nanopor sp.#6	Nanopor sp.#7	Nanopor sp.#8
MCu-01	Vinales	Ulidiidae	2	HG		*Kentomonas* sp. 1a								
MCu-02	Vinales	Muscidae	2	HG+MG	*Herpetomonas* sp. 2		*Vickermania* sp. 8	*Vickermania* sp. 13	“Muscomonas”	“Newbiana”	*Herpetomonas* sp. 1	*Herpetomonas* sp. 2	*Herpetomonas* sp. 3	*Angomonas* sp. 1
MCu-03	Vinales	Muscidae	2	MG (?)			*Vickermania* sp. 8	*Vickermania* sp. 11	*Vickermania* sp. 14	Vickermania sp. 15	“Muscomonas”			
MCu-04	Vinales	Muscidae	2	MG (?)	*Wallacemonas* sp. A		*Vickermania* sp. 8	*Vickermania* sp. 11	*Vickermania* sp. 15	“Muscomonas”				
MCu-05	Vinales	Muscidae	3	MG	*Wallacemonas* sp. A		*Vickermania* sp. 8	*Vickermania* sp. 9	*Vickermania* sp. 14	Vickermania sp. 15	“Muscomonas”			
MCu-06	Vinales	Ulidiidae	2	HG	*Wallacemonas* sp. A	*Vickermania* sp. 2a								
MCu-07	Vinales	Sepsidae	3	MG			*Vickermania* sp. 10	*Parabodo caudatus*						
MCu-08	Vinales	Sepsidae	3	MG			*Vickermania* sp. 2b	*Vickermania* sp. 7	*Vickermania* sp. 10					
MCu-09	Vinales	Sepsidae	3	MG		*Vickermania* sp. 1								
MCu-10	Vinales	Sepsidae	3	MG			*Vickermania* sp. 10	*Parabodo* sp. 1	*Parabodo* sp. 2	*Parabodo* sp. 3				
MCu-11	Vinales	Sepsidae	3	MG										
MCu-12	Palma Rubia	Muscidae	2	MG			*Vickermania* sp. 6	*Vickermania* sp. 8	*Vickermania* sp. 9	*Vickermania* sp. 11	*Vickermania* sp. 12	*Vickermania* sp. 15	“Muscomonas”	“Newbiana”
MCu-13	Palma Rubia	Muscidae (cf.)	2	HG+MG	*Herpetomonas modestus*		*Vickermania* sp. 16	*Herpetomonas modestus*						
MCu-14	Palma Rubia	Muscidae	4	MG			*Vickermania* sp. 6	*Vickermania* sp. 9	*Vickermania* sp. 12	*Vickermania* sp. 15	“Muscomonas”			
MCu-15	Cienfuegos	Lauxaniidae (cf.)	3	HG+MG		*Vickermania* sp. 4								
MCu-16	Trinidad	Calliphoridae	3	HG+MG	*Angomonas* sp. 1	*Vickermania* sp. 3								
MCu-17	Varaderos	Muscidae	3	MG		*Vickermania* sp. 5								
MCu-18	Varaderos	Muscidae	3	HG		*Kentomonas* sp. 1b								
MCu-19	Varaderos	Muscidae	2	HG	*Kentomonas* sp. 1a		*Kentomonas* sp. 1a	*Herpetomonas samuelpessoai*						
MCu-KV	Varaderos	Drosophilidae	1	?		*Wallacemonas* -like								

* Intensity of infection (1-4)
** Localization in host: HG, hindgut; MG, midgut
